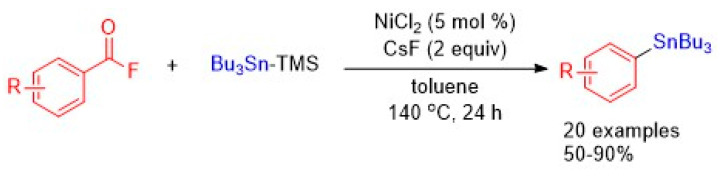# Editorial for “Organometallic Chemistry” Section, in Journal *Molecules*

**DOI:** 10.3390/molecules25133038

**Published:** 2020-07-03

**Authors:** Michal Szostak

**Affiliations:** Department of Chemistry, Rutgers University, Newark, NJ 07102, USA; michal.szostak@rutgers.edu

It is a great pleasure to assume the role of Editor-in-Chief for the “Organometallic Chemistry” Section of *Molecules* (https://www.mdpi.com/journal/molecules/sections/organometallic_chemistry). Organometallic chemistry has always taken a front seat among the chemical disciplines. All chemists and students in chemistry are well familiar with the landmark organometallic discoveries that have absolute utility across all areas of chemistry. From the Grignard addition that is introduced in undergraduate curricula as the fundamental carbon–carbon bond-forming reaction—whose discoverer was awarded the Nobel Prize in Chemistry in 1912—through to Ziegler–Natta polymerization using Al, Ti or Zr; the discovery of sandwich compounds by Wilkinson and Fischer; the colorful history of Woodward’s true input in elucidating the structure and properties of ferrocene; the remarkable contributions to asymmetric synthesis by Knowles, Noyori and Sharpless using Rh, Ru and Ti; the essential studies of magic alkene metathesis by Chauvin, Grubbs and Schrock relying on Mo and Ru; and the cross-coupling reactions discovered by Heck, Negishi and Suzuki using Pd, to name just a few, all of these processes have been the key driving force for other chemical disciplines and provided the golden benchmark to which all new transformations and processes should aspire.

The “Organometallic” Section of *Molecules* strives to provide an open-access platform for disseminating high quality articles and reviews at the core of organometallic chemistry. In this respect, the “Organometallic” Section invites studies on the synthesis of organometallic complexes, catalysis, catalytic processes, bioorganometallic chemistry, the structure and reactivity of main group metals, transition metals, and lanthanides, among other subfields of organometallic chemistry. Furthermore, the “Organometallic” Section welcomes research articles and reviews at the interface of organometallic chemistry and organic and inorganic chemistry, as well as those regarding all aspects of organometallic chemistry, ranging from synthesis to application.

Historically, organometallic research has been published in *Organometallics* and *Dalton Transactions*, the flagship journals of the American Chemical Society and the Royal Society of Chemistry, respectively. Other popular outlets include *J. Organomet. Chem.* and *Eur. J. Inorg. Chem.*, published by Elsevier and Wiley. With the advent of open-access publishing, *Molecules* and MDPI have been steadily growing in recognition and influence among the chemical community, and this year, the journal celebrates its 25-year anniversary. 

The statistical data for *Molecules* compare very favorably with those for the competing journals in the field. The journal’s impact factor of 3.3 ranks *Molecules* as one of the highest among the organometallic journals. For the sake of comparison, the impact factors of *Organometallics* (3.8) and *Dalton Transactions* (4.2) are slightly higher, as expected for the leading organometallic journals of the ACS and RSC, while those of *J. Organomet. Chem.* (2.3) and *Eur. J. Inorg. Chem.* (2.5) are lower. Furthermore, the total number of downloads/views of manuscripts published in *Molecules* in 2019 was 4,746,115, which, for the total of 4619 articles published, implies approximately 1000 views per article, attesting to superb visibility among both researchers and the community. Finally, in terms of publication time, *Molecules* is among the fastest in the field, with an average of 32 days from submission to first publication. The rapid publishing time, while maintaining a rigorous peer-review process, is undeniably a credit to the dedication and professionalism of the Editors and the Editorial Team of *Molecules*. 

It is clear that (1) a favorable and steadily increasing impact factor (IF = 3.3), (2) high visibility in open-access publishing, (3) rapid publication times, and (4) the high quality of the rigorous peer-review process are among the benefits of publishing in the “Organometallic” Section of *Molecules*. There are also additional benefits. (1) We encourage authors and readers to take advantage of Special Issues that focus on a specific area of organometallic chemistry and enjoy record visibility and higher readership than regular issues. (2) To celebrate the 25th anniversary of *Molecules*, we will launch a Virtual Issue highlighting the high-caliber organometallic research published in the “Organometallic” Section. A selection of cutting-edge “Hot-Papers” published in the Organometallic Section of *Molecules* is presented at the end of this Editorial [1,2,3,4,5,6,7,8,9,10]. (3) Finally, in the era of modern publishing with on-the-go mobile access, the Editorial Team at *Molecules* ensures that the published research enjoys rapid dissemination through a variety of platforms to reach everyone and maximize access. This includes Twitter @Molecules_MDPI, Facebook and LinkedIn with the launch of regular highlights of “Hot Papers” and “Editors’ Choice” manuscripts. 

Organometallic chemistry is experiencing rapid progress. With the improvements in characterization and analytical techniques, the development of new technologies and the continuous expansion of the carbon-metal toolbox, the prospective contribution of organometallic research to chemistry seems limitless. We hope that you will consider submitting your organometallic papers to *Molecules*.

**When submitting organometallic papers to *Molecules*, “Organometallic Chemistry Section” should be selected in the scroll-down submission menu.**

**“Hot Articles” in the Organometallic Section of *Molecules***

**1. Recent Developments in the Medicinal Applications of Silver-NHC Complexes and Imidazolium Salts**
  by Nicholas A. Johnson, Marie R. Southerland and Wiley J. Youngs
  *Molecules* 2017, 22(8), 1263; https://doi.org/10.3390/molecules22081263

**2. First-Row Late Transition Metals for Catalytic Alkene Hydrofunctionalisation: Recent Advances in C-N, C-O and C-P Bond Formation**
  by Sophie Bezzenine-Lafollée, Richard Gil, Damien Prim and Jérôme Hannedouche
  *Molecules* 2017, 22(11), 1901; https://doi.org/10.3390/molecules22111901

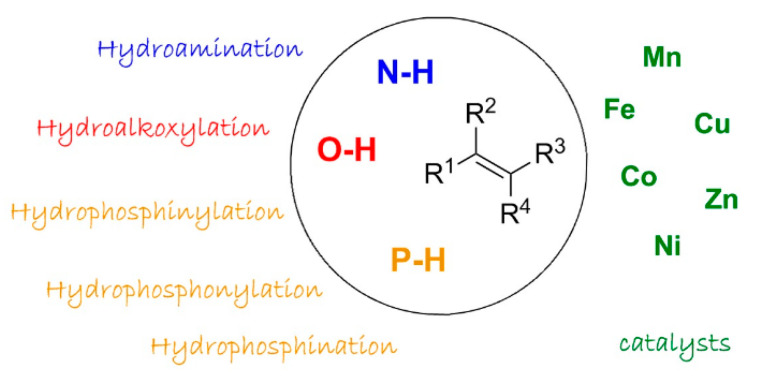

**3. NAMI-A and KP1019/1339, Two Iconic Ruthenium Anticancer Drug Candidates Face-to-Face: A Case Story in Medicinal Inorganic Chemistry**
  by Enzo Alessio and Luigi Messori
  *Molecules* 2019, 24(10), 1995; https://doi.org/10.3390/molecules24101995

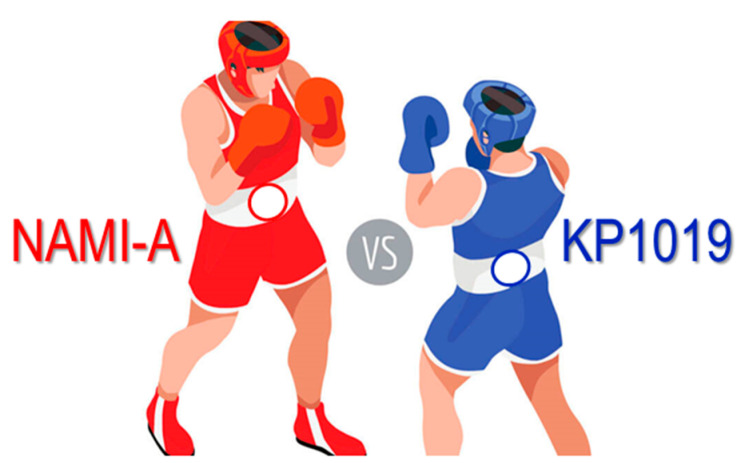

**4. Novel Cage-Like Hexanuclear Nickel(II) Silsesquioxane. Synthesis, Structure, and Catalytic Activity in Oxidations with Peroxides**
  by Alexey N. Bilyachenko, Alexey I. Yalymov, Lidia S. Shul’pina, Dalmo Mandelli, Alexander A. Korlyukov, Anna V. Vologzhanina, Marina A. Es’kova, Elena S. Shubina, Mikhail M. Levitsky and Georgiy B. Shul’pin
  *Molecules* 2016, 21(5), 665; https://doi.org/10.3390/molecules21050665

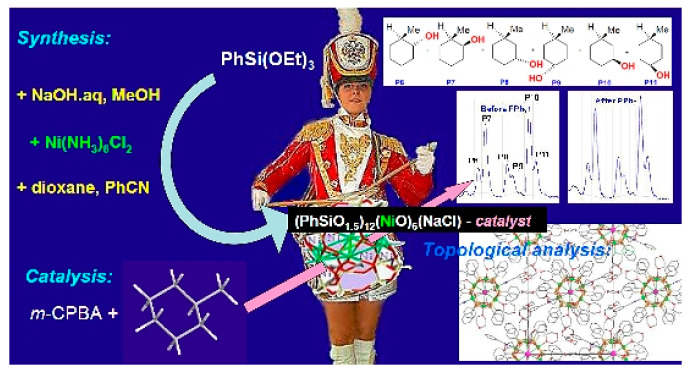

**5. Lewis Pair Catalysts in the Polymerization of Lactide and Related Cyclic Esters**
  by Xinlei Li, Changjuan Chen and Jincai Wu
  *Molecules* 2018, 23(1), 189; https://doi.org/10.3390/molecules23010189

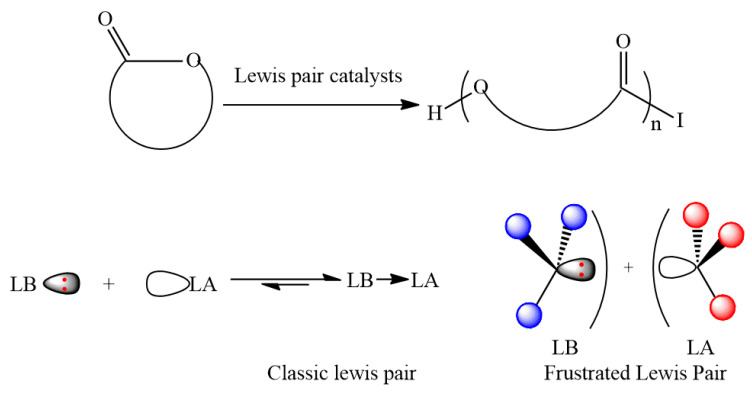

**6. The Lewis Pair Polymerization of Lactones Using Metal Halides and N-Heterocyclic Olefins: Theoretical Insights**
  by Jan Meisner, Johannes Karwounopoulos, Patrick Walther, Johannes Kästner and Stefan Naumann
  *Molecules* 2018, 23(2), 432; https://doi.org/10.3390/molecules23020432

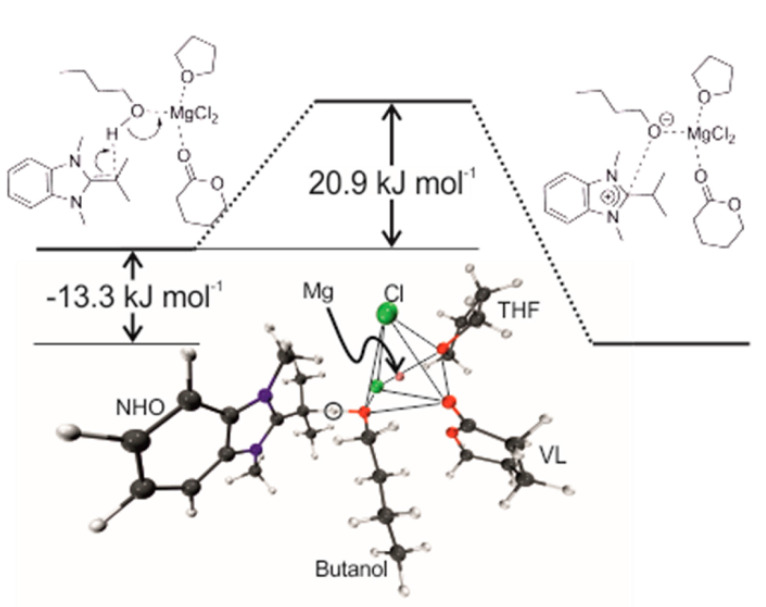

**7. Dinuclear Nickel(I) and Palladium(I) Complexes for Highly Active Transformations of Organic Compounds**
  by Takahiro Inatomi, Yuji Koga and Kouki Matsubara
  *Molecules* 2018, 23(1), 140; https://doi.org/10.3390/molecules23010140

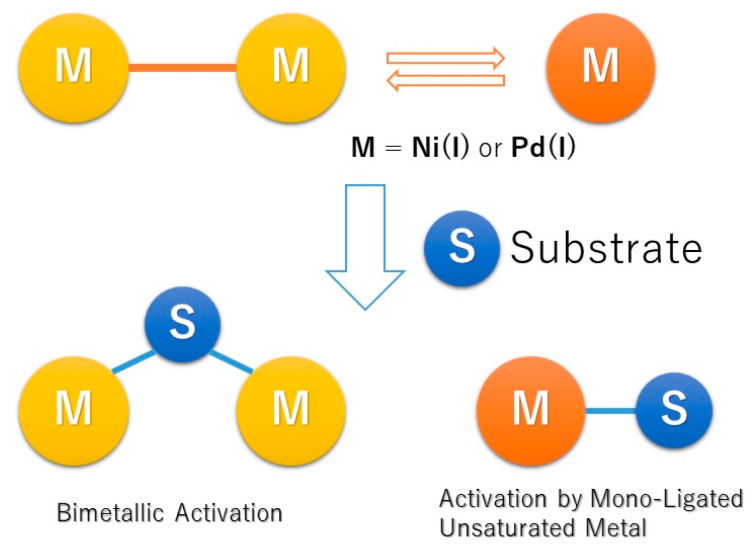

**8. Polysiloxane/Polystyrene Thermo-Responsive and Self-Healing Polymer Network via Lewis Acid-Lewis Base Pair Formation**
  by Fernando Vidal, Huina Lin, Cecilia Morales and Frieder Jäkle
  *Molecules* 2018, 23(2), 405; https://doi.org/10.3390/molecules23020405

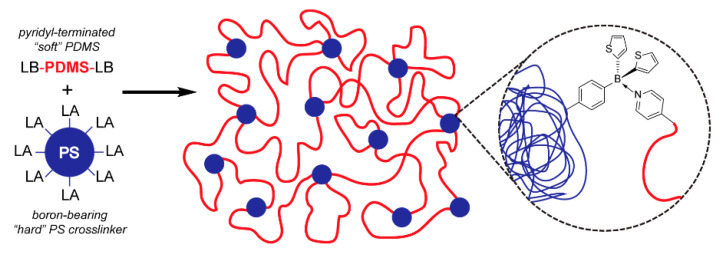

**9. Highly Linear Polyethylenes Achieved Using Thermo-Stable and Efficient Cobalt Precatalysts Bearing Carbocyclic-Fused NNN-Pincer Ligand**
  by Jingjing Guo, Zheng Wang, Wenjuan Zhang, Ivan I. Oleynik, Arumugam Vignesh, Irina V. Oleynik, Xinquan Hu, Yang Sun and Wen-Hua Sun
  *Molecules* 2019, 24(6), 1176; https://doi.org/10.3390/molecules24061176

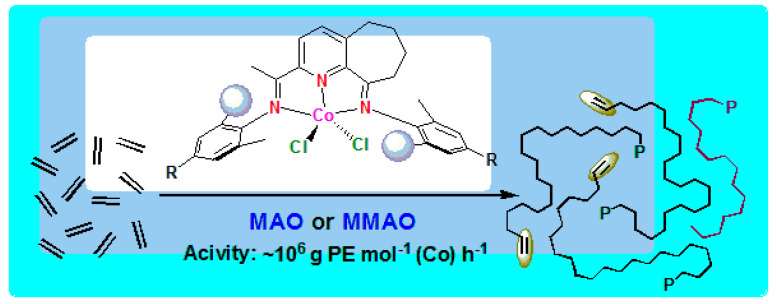

**10. Nickel-Catalyzed Decarbonylative Stannylation of Acyl Fluorides under Ligand-Free Conditions**
  by Xiu Wang, Zhenhua Wang, Li Liu, Yuya Asanuma and Yasushi Nishihara
  *Molecules* 2019, 24(9), 1671; https://doi.org/10.3390/molecules24091671